# Cultivation substrates alter plant metabolic profiles with concomitant impacts on herbivore performance

**DOI:** 10.3389/fmicb.2026.1893579

**Published:** 2026-09-01

**Authors:** Shimei Huo, Kang Xie, Lei Chen, Jingtao Sun, Shan Wang

**Affiliations:** 1 Department of Agronomy and Horticulture, Jiangsu Vocational College of Agriculture and Forestry, Zhenjiang, China; 2 Hubei Key Laboratory of Regional Development and Environmental Response, Faculty of Resources and Environmental Sciences, Hubei University, Wuhan, China; 3 State Key Laboratory of Agricultural and Forestry Biosecurity, College of Plant Protection, Nanjing Agricultural University, Nanjing, China

**Keywords:** cultivation substrate, feeding performance, microbiome, secondary metabolites, spider mite

## Abstract

**Introduction:**

Herbivore microbiomes are strongly influenced by host plant quality and diet composition. However, it remains unclear how different cultivation substrates modulate the plant metabolome and how these shifts subsequently influence the performance and microbiome of aboveground herbivores.

**Methods:**

We utilized common beans cultivated in soil and hydroponic systems to investigate the multi-trophic interactions involving the substrate microbiome, plant metabolome and the polyphagous spider mite *Tetranychus pueraricola*.

**Results:**

Our results showed that while *T. pueraricola* exhibited an increased oviposition on hydroponic leaves, they achieved markedly higher biomass when reared on soil-grown common beans. The beta diversity of the mite microbiome differed markedly between two systems and showed close associations with substrate microbial composition. Metabolomic profiling revealed significant enrichment in isoflavonoid biosynthesis and carbon metabolism pathways between hydroponic and soil-grown leaves. Specifically, the upregulation of key secondary metabolites in hydroponic leaves correlated with suppressed mite weight. Comprehensive correlation analyses further indicated that cultivation substrate affected multi-scale associations among substrate microbiome and leaf metabolome and mite microbiome.

**Discussion:**

Our results demonstrate that growth substrates potentially contribute to divergence in herbivore microbiome and performance, as well as plant metabolism. These findings highlight the significant influence of cultivation substrate on aboveground plant-herbivore interactions, providing new insights for optimizing crop management and ecological pest control.

## 1 Introduction

Herbivores have developed intricate associations with microbial communities through long-term coevolution in natural ecosystems, in which herbivore-associated microbiomes play key roles in modulating host development, immunity, defense against abiotic and biotic stresses (Holt et al., 2024; Kaltenpoth et al., 2025). The feeding behavior of

herbivorous insects maintains a dynamic equilibrium with plants and their growth environments, herbivore microbiomes are consequently susceptible to the influences exerted by host plants and environmental factors, such as cultivation substrates. Thus, understanding whether variation in herbivore microbiome primarily driven by the host plant or other environmental drivers has received considerable attention. Recent studies have shown that environmental factors significantly shape the composition of insect gut microbiota (Muratore et al., 2020). For instance, Hannula et al. (2019) demonstrated that the microbiome of foliar-feeding insects are not derived from consumed diets but direct from the soil. Previous studies have demonstrated that the soil or plant growth substrate microbiome can directly alter herbivore microbiomes and indirectly influence them by mediating changes in the plant microbiome and host nutritional quality (Kikuchi et al., 2007; Hannula et al., 2019; Santos-Garcia et al., 2020), however, further evidence is still needed to elucidate how changes in plant growth substrates modulate the interactions between aboveground plants and their herbivores.

There is a complex interplay among plants, herbivores and plant growth environment in natural ecosystems, how the physicochemical and biological properties of the soil substrate affect aboveground plant nutrition, defensive metabolites and herbivore feeding performance remains a fundamentally important question for agricultural systems (Bezemer and van Dam, 2005). Recent evidence has revealed that plant chemical composition and metabolism are modulated by nutrient availability in different cultivation systems (Altieri and Nicholls, 2003) and by soil microbial characteristics (Huberty et al., 2022). Such alterations in plant traits subsequently influence the behavioral and physiological responses of herbivores to soil legacies (Kos et al., 2015; Kostenko et al., 2012). Plant secondary metabolites, such as phenolics, terpenoids, steroids, alkaloids and flavonoids, play crucial roles in plant growth and development, defense responses and adaptation to various stresses, and also function as key mediators in plant-microbe interactions (Pang et al., 2021). For example, plants can recruit beneficial rhizosphere *Pseudomonas* by modulating flavonoid synthesis to enhance nitrogen acquisition in nutrient-deficient environments (Wu et al., 2025). Other studies have shown that root-exuded flavonoids and isoflavones serve as key chemoattractants for rhizobia, thereby promoting the establishment of legume-rhizobia symbiosis (Hartman et al., 2017). In fact, the physiological condition of herbivores depends directly and indirectly on the traits of the plant tissue they feed upon (Mattson, 1980; Ohgushi, 2005), and microbiomes associated with growth substrates or host plants can modulate herbivorous insect behavior in both positive and negative ways by affecting plant metabolite profiles. For instance, volatile emissions from fungus-infected plants are detected by herbivores as chemical indicators of suitable foraging or oviposition sites Xu et al., (2023). Harmsen et al. (2024) further revealed that plants grown in the most suppressive soil enriched with plant-beneficial bacteria promoted plant resistance against the herbivorous pest. Beyond these dynamics, gut microbiota also play the pivotal roles in plant-herbivore interaction, including nutrient digestion and toxin degradation. The endosymbiont *Stammera* enables the sap-sucking beetle *Cassida rubiginosa* to acquire pectin from plant cell wall by secreting pectinases (Salem et al., 2017). Gut-associated *Pseudomonas* bacteria of the coffee berry borer *Hypothenemus hampei* enables the host to neutralize plant-derived toxic alkaloids by relying entirely on nitrogen and carbon sources from the degradation of plant caffeine (Ceja-Navarro et al., 2015). Remarkably, nitrogen-fixing bacteria, as key members of the insect microbiome, are critical for modulating host nutritional requirements (Bar-Shmuel et al., 2020). Previous studies have found that nitrogen fixation by gut symbionts is important for the survival of termites (Brune, 2014). Additionally, several studies have demonstrated that *Candidatus* Dactylopiibacterium carminicum, identified as a nitrogen-fixing symbiont in carmine cochineal *Dactylopius* coccus, may compensate for nitrogen deficiencies in the insect's diet and provide essential amino acids to the host (Vera-Ponce de León et al., 2017; Rosenblueth et al., 2018).

Advancements in agricultural cultivation have intensified the complexity of plant and substrate interactions. Previous studies have primarily focused on how soil or vermiculite-based cultivation systems modulate nutrient accumulation, secondary metabolites in plant tissues, and the resulting herbivore microbial communities and performance (Altieri and Nicholls, 2003; Yuan et al., 2023; Hannula et al., 2019; Pineda et al., 2020). Notably, Yuan et al. (2023) have established a well-validated regulatory paradigm for substrate-mediated plant-chewing herbivore interactions, showing that cultivation substrates reshape aboveground plant metabolomes and microbiomes and thereby modulate herbivore performance and symbiotic microbiota. However, it remains unknown whether this pattern extends to piercing-sucking herbivores in hydroponic systems, and associations between substrate microbial communities and those of aboveground herbivores remain unquantified. Building on this prior framework, we investigated the piercing-sucking spider mite *Tetranychus pueraricola* using a hydroponic and soil cultivation contrast. Specifically, we profiled the leaves metabolomes of common bean (*Phaseolus vulgaris* L.), microbiomes of growth substrates and spider mite to assess how cultivation conditions influence shifts in herbivore fitness (body weight and fecundity) and microbial community. We further carried out cross-omics associations across cultivation systems to disentangl correlation between microbiota and plant metabolism, expanding the current framework for understanding how belowground factors are linked to aboveground plant-herbivore-microbe interactions.

## 2 Materials and methods

### 2.1 Sampling and preference bioassay

The spider mite *Tetranychus pueraricola* in this study was originally collected from Huzhou of Zhejiang Province in 2023. The species was identified based on a combination of morphological characteristics and molecular data. Morphological identification from a glass slide of aedeagus of their male mites; the molecular identifications were performed according to the sequences of ribosomal gene ITS and mitochondrial gene COI by using primers primers rDO2 (5′-GTCGTAACAAGGTTTCCGTAGG −3′) and HC2 (5′-ATATGCTTAAGTTCAGCGGG −3′) for ribosomal ITS, and primers COI-F (5′-AAGAGGAGGAGGAGACCCAATT −3′) and COI-R (5′-AAACCTCTAAAAATAGCGAATACAGC −3′) for mitochondrial gene COI, respectively. This population was then reared on common bean (*Phaseolus vulgaris* L.) leaves at 25 ± 1 °C, 60%−70% relative humidity (RH) and a 16:8-h (light/dark) photoperiod for nearly a year prior to the experiments. To monitor responses to the common bean grown in different cultivation substrates, we conducted a series of feeding bioassays. Common bean seedlings at 10 days post-sowing were subjected to two treatments: each plant was grown in a separate plastic cup under either hydroponic cultivation (with 1 mL of nutrient solution added to 200 mL of ultrapure water) or soil substrate cultivation (supplied with 100 g of the same seedling-raising soil). Hydroponic nutrient solutions specifically formulated for vegetable cultivation by China Agricultural University were used in this study. Fecundity and body weight of *T. pueraricola* were evaluated after standardized substrate treatment of the host plants. Common bean plants in both assays had experienced exactly 17 days of substrate treatment at the time of final data collection.

For the body weight assay, plants were treated for 2 days before 120 mated adult female mites were placed on the leaves for feeding and oviposition. Females were removed after 12 h, and their eggs were left to develop on the plants. These eggs developed into 2-day-old virgin adult females in approximately 15 days. At the time of collection, the plants had received a total of 17 days of treatment. For each replicate, 100 two-day-old virgin females were collected and weighed using a microbalance (Ohaus, AR223CN). Additionally, 50 females per replicate were used for bacterial 16S rRNA gene amplicon sequencing. For the fecundity assay, 20 newly emerged adult females per replicate were placed on plants that had already been treated for 14 days. Their fecundity was recorded over 3 days. At the end of the recording period, the plants had also received a total of 17 days of treatment. Each treatment consisted of six replicates, and lanolin was applied to the base of the petioles on all plants to prevent mite escape. Statistical analyses were performed using GraphPad Prism v9.5.1. Data normality was assessed using the Shapiro-Wilk test. As the data followed a normal distribution and exhibited homogeneity of variances (*F*-test), differences in mite fitness between the soil and hydroponic systems were analyzed using two-tailed unpaired *t*-test, and *P* < 0.05 was considered statistically significant.

### 2.2 Plant, soil and water sampling for microbiome analysis

To evaluate the influence of plants or cultivation substrate on spider mite bacterial communities, samples of plant roots, rhizosphere soil, and hydroponic water were collected from each system. Six independent biological replicates were performed for each sample. Root samples were transferred to 15 mL centrifuge tubes filled with 10 mL of phosphate-buffered saline (1 × PBS), followed by ultrasonic treatment for 5–10 min, and then stored at −80°C after rinsing three times with autoclaved demineralized water and flash-frozen in liquid nitrogen. Rhizosphere soils from soil-grown plant were obtained by first removing the bulk soil by shaking, the remaining soils then were gently collected in sterile tube and stored at −80°C until processing. For water sample collection, 1,000 mL of nutrient solution from the root zone of the hydroponic system was filtered through sterile 0.22 μm membrane filters, and the filters were subsequently stored at −80°C.

### 2.3 Sample DNA extraction and amplicon sequencing

Before DNA extraction, pooled adult female mites were rinsed in 75% ethanol for 30 s and washed three times with sterile ultrapure water to remove cuticular and environmental contaminants. Total genomic DNA was extracted from all samples employing the CTAB-based DNA extraction protocol (Wesolowska-Andersen et al., 2014). The purity and concentration of DNA were assessed using 1.0% agarose gel electrophoresis, the qualified samples were used as DNA templates to amplify the 16S V4 region with primers 515F and 806R (Walters et al., 2016). All PCR reactions were carried out with 15 μL of Phusion^®^ High-Fidelity PCR Master Mix (New England Biolabs), 0.2 μM of forward and reverse primers, and about 10 ng DNA template. Amplification was performed with an initial denaturation at 98 °C for 1 min, followed by 30 cycles of denaturation at 98 °C for 10 s, annealing at 50 °C for 30 s, elongation at 72 °C for 30 s, and a final extension at 72 °C for 5 min. After mixing and purification of PCR products, sequencing libraries were generated and indexes were added. The library was assessed using Qubit and real-time PCR for quantification, and bioanalyzer for size distribution analysis. Quantified libraries were pooled and sequenced on Illumina NovaSeq PE250 based on effective library concentration and data amount required.

### 2.4 Species annotation and microbial diversity analyses

Paired-end reads were assigned to samples based on their unique barcode, after trimming the barcode and primer sequences, reads from each sample were merged using min-overlap parameter of FLASH v1.2.11 to generate raw tags (Magoč and Salzberg, 2011). Quality filtering on the raw tags was carried out using parameter -q of the fastp v0.23.1 to obtain high-quality clean reads (Bokulich et al., 2013). Chimeric sequences were detected and removed by aligning the clean tags against SILVA database (https://www.arb-silva.de/), resulting in the final effective tags (Edgar et al., 2011). These reads were then subjected to denoising using DADA2 module in the QIIME2 v202202 to generate the final amplicon sequence variants (ASVs) (Callahan et al., 2016). Species annotation was performed using QIIME2 against the SILVA database, and taxonomic abundance profiles were generated based on the ASV annotations (Bokulich et al., 2018; Bolyen et al., 2019).

Alpha diversity analysis was performed to estimate diversity and richness of the microbial community within individual samples, with the Chao1 index calculated using QIIME2. Principal Coordinate Analysis (PCoA), which serves to extract principal coordinates and visualize patterns within complex datasets (Minchin, 1987), was conducted based on weighted UniFrac distances using the ade4 and ggplot2 packages in R v4.0.3 (Dray and Dufour, 2007; Wickham, 2011). To assess whether differences in beta diversity were driven by shifts in community centroids or by differences in within-group dispersion, PERMDISP (betadisper) tests were performed using the betadisper function in the R vegan package. Ternary plots were generated to compare bacterial community composition among spider mites, cultivation substrates and plant roots using R ggtern package (Hamilton and Ferry, 2018). To determine whether the substrate-dependent spider mite microbiota is more similar to the substrate microbiota or the plant root microbiota, the proportion of mite-specific ASVs shared with substrates and shared with roots in each cultivation substrate were calculated, bar plots were generated using the ggplot2 package in R.

### 2.5 Metabolomes of leaf samples

Common bean plants were cultivated under soil or hydroponic systems for 17 days before leaf collection. Six biological replicates were sampled for each substrate treatment, with each sample weighing 100 mg. The leaves were finely ground in liquid nitrogen, and the homogenate was subsequently resuspended in prechilled 80% methanol via thorough vortexing. The mixture was incubated on ice for 5 min and then was centrifuged at 15,000 × g and 4°C for 20 min. A portion of the supernatant was diluted to final concentration containing 53% methanol by LC-MS-grade water. The samples were subsequently transferred to a new Eppendorf tube and then were centrifuged under the same conditions (15,000 × g, 4°C, 20 min). The resulting supernatant was collected and subjected to LC-MS analysis (Want et al., 2013). Quality control (QC) samples were prepared by pooling equal aliquots from all biological samples. Three pooled QC samples were injected before sample analysis to assess instrument performance and equilibrate the LC-MS system. Another three QC samples were analyzed for segmented MS/MS acquisition to facilitate metabolite identification in combination with the experimental samples. During sample analysis, QC samples were injected at regular intervals to monitor system stability and support data quality control.

UHPLC-MS/MS analyses were performed on a Vanquish UHPLC system (Thermo Fisher Scientific, Germany) coupled with an Orbitrap Q Exactive™ HF mass spectrometer (Thermo Fisher Scientific, Germany). Samples were injected onto a Hypersil Gold column (100 × 2.1 mm, 1.9 μm) and separated using a 12-min linear gradient at a flow rate of 0.2 mL per minute. Eluent A (0.1% formic acid in water) and eluent B (methanol) were used as the mobile phases for positive and negative polarity modes, the gradient program was set as follows: 2% B (1.5 min), 2–85% B (3 min), 85–100% B (10 min), 100–2% B (10.1 min), and 2% B (12 min). The raw data files generated by UHPLC-MS/MS were processed using the Compound Discoverer 3.3 (Thermo Fisher Scientific) for peak alignment, peak detection, metabolite quantification, and normalization. Peak intensities were normalized to the total spectral intensity. The normalized data was used to predict the molecular formula based on additive ions, molecular ion peaks and fragment ions, and peaks were then matched with the mzCloud (https://www.mzcloud.org/), mzVault and MassList database to obtain the accurate qualitative and relative quantitative results. Metabolites identified in positive and negative ionization modes were integrated into a unified metabolite dataset after removal of redundant metabolite features for subsequent statistical analyses.

Metabolites were annotated using the KEGG database (https://www.genome.jp/kegg/pathway.html), HMDB database (https://hmdb.ca/ metabolites) and LIPIDMaps database (http://www.lipidmaps.org/). Principal components analysis (PCA) and Partial least squares discriminant analysis (PLS-DA) were performed with stats and ropls packages. PLS-DA model performance was evaluated using seven-fold cross-validation, and the goodness-of-fit (R^2^Y) and predictive ability (Q^2^Y) were calculated. Model overfitting was further assessed using a 200-permutation test. A negative *Q*^2^ intercept and higher *R*^2^ values than *Q*^2^ values were considered indicative of a valid model without over fitting. Screening of differential metabolites was primarily based on three parameters: variable importance in the projection (VIP) values, fold change (FC) of metabolites between the two groups, and FDR-adjusted *P* values. Raw *P* values were adjusted for multiple testing using the Benjamini-Hochberg (BH) procedure implemented with the p.adjust function in R. The metabolites with VIP > 1, adjusted *P* < 0.05, and FC ≥ 1.5 or FC ≤ 0.7 were considered to be significantly differential metabolites. Volcano plots were generated based on log2 (Fold Change) and -log10 (*p*-value) of metabolites by ggplot2 in R. Functional annotation and metabolic pathways were conducted based on the KEGG database. KEGG pathway enrichment analysis of the differential metabolites was performed using a hypergeometric test. Multiple-testing corrections were applied using the Benjamini-Hochberg (BH) adjusted method. To capture comprehensive biological insights in this untargeted metabolomic profile, pathways with an unadjusted *P* < 0.05 were selected for downstream biological discussion, while their corresponding FDR-adjusted *P* values are provided transparently in the supplementary data.

### 2.6 Correlation analyses

To explore cross-domain associations among the substrate microbiome, leaf metabolome, and mite microbiome, we performed Procrustes analysis on PCA score configurations (axes retained to explain ≥ 50% cumulative variance) and Mantel or partial Mantel tests on pairwise Aitchison (microbiome) or Euclidean (metabolite) distance matrices, with partial tests conditioning on either the third omics layer or the cultivation group. To identify candidate metabolites associated with microbial community structure, we performed Random Forest regression using the rfPermute package in R (500 trees), with metabolite abundances as predictors and the first principal coordinate axis of microbial Bray-Curtis distances as the response. The top 30 metabolites explaining substrate microbiome structure and mite microbiome structure were selected for downstream analysis. Mantel tests were then performed to evaluate the correlation between the metabolite distance matrix and each microbiome distance matrix. The top 15 metabolites ranked by the sum of absolute Mantel r values were visualized using the linkET package in R (Huang, 2021). To identify metabolites linked to mite fitness, we first applied Random Forest regression (rfPermute, 500 trees) with within-group Wilcoxon rank-sum filtering (FDR < 0.05) to select candidate metabolites, then computed Spearman correlations between each candidate and fitness traits (body weight and fecundity) separately within each cultivation group. Because no metabolite reached FDR < 0.05 and |ρ| ≥ 0.6 for either trait, bubble plot were generated based on *P* values. Pearson correlations were computed between all pairs of substrate ASVs and mite ASVs using the cor. test function in R. Because the within-system sample size was only six replicates, we therefore selected the top 40 candidate pairs ranked by absolute value of r in each system for visualization. The overall relationship between substrate and mite microbiome community compositions was assessed by Mantel tests with 999 permutations using Spearman correlation on Bray-Curtis distance matrices. Network visualization was generated using the igraph package in R.

## 3 Results

### 3.1 Impacts of cultivation systems on spider mite fitness and microbiome

**Figure 1:**
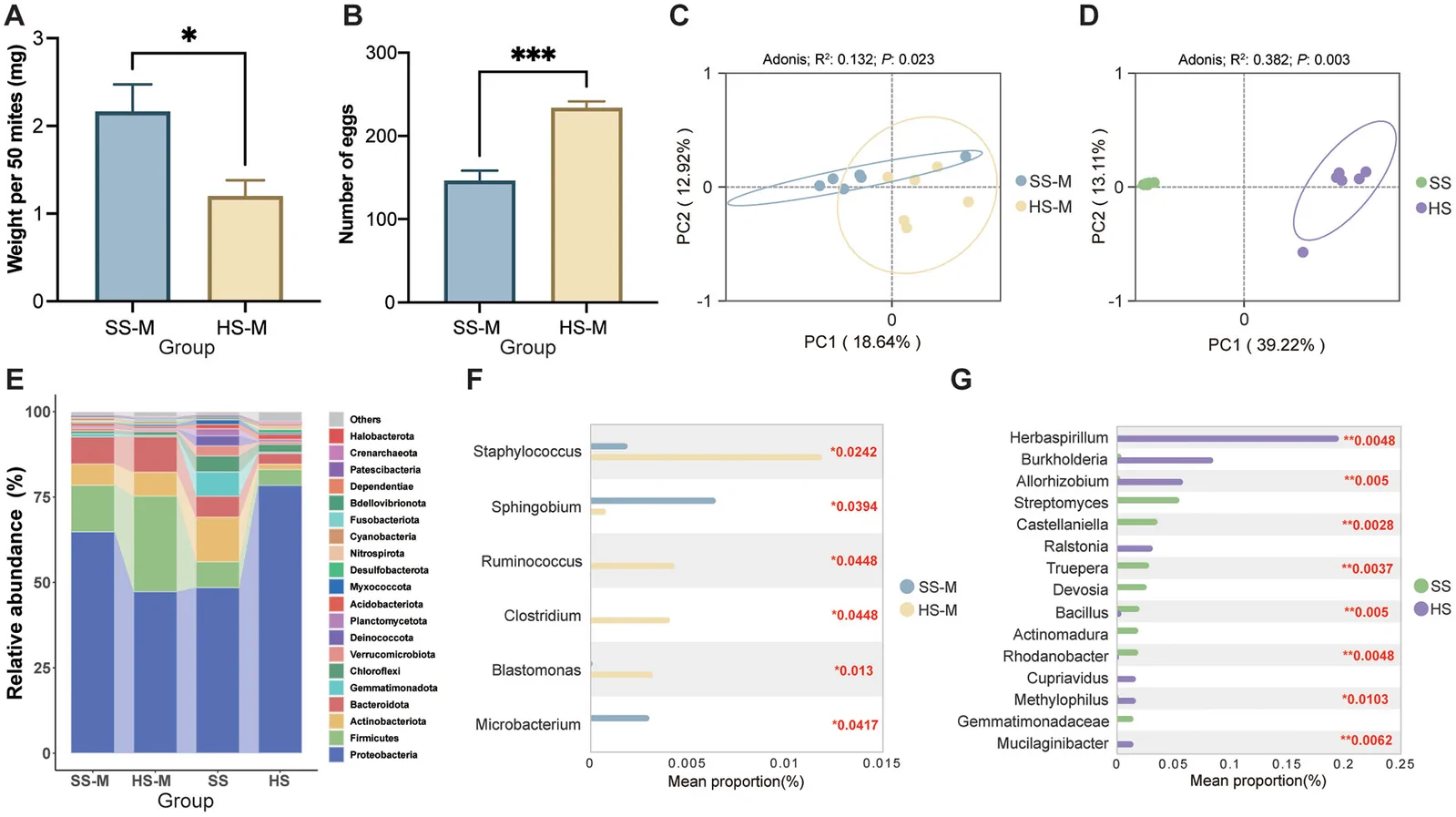
Fitness and microbial communities of spider mite *T. pueraricola* feeding on common bean plants grown in soil and hydroponics systems. **(A)** Body weight of 2-day-old virgin females. (**B)** Number of eggs laid per replicate over 3 days by 1-day-old adult females (*n* = 20 per replicate). Results are presented as mean ± SEM and differences were analyzed by unpaired *t* test (*P < 0.05; **P < 0.01; ***P < 0.001). **(C, D)** Principal coordinate analysis (PCoA) based on weighted UniFrac distances of microbiome associated with the spider mites **(C)** and cultivation substrates **(D)** under different systems. *R*^2^ and *P* values are based on an Adonis test. **(E)** Relative abundance (%) of the top 20 major phyla in bacterial communities of spider mites and different cultivation substrates. (**F, G)** Significant genus-level variations in bacteria between spider mites **(F)**and substrates **(G)** under different cultivation systems based on the Wilcoxon rank-sum test. SS-M, spider mite *T. pueraricola* in soil system; HS-M, spider mite *T. pueraricola* in hydroponics system; SS, soil substrate; HS, hydroponic substrate.

To evaluate the impacts of different cultivation systems on the performance of spider mites, we compared the body weight and fecundity of spider mites reared on soil-grown and hydroponic plants. Compared to those in the soil system, spider mites feeding on leaves from the hydroponic system exhibited a significant reduction in body weight (unpaired *t* test: *t* = 2.711, df = 10, *P* = 0.019; Figure 1A), yet a marked increase in oviposition (*t* = 6.180, df = 10, *P* < 0.001; Figure 1B). Bacterial alpha diversity within the spider mite microbiome did not differ significantly between the two cultivation systems, (Supplementary Figure 1A in Supplementary Information), whereas beta diversity was distinctly altered by the hydroponic vs soil substrate (Figure 1C), and this difference was not explained by differences in multivariate dispersion (PERMDISP, *P* > 0.05). Notably, this divergence in mite-associated microbes mirrored the shifts observed in the microbial communities of their respective cultivation substrates (Figure 1D; Supplementary Figure 1B in Supplementary Information). PERMDISP indicated significant differences in multivariate dispersion (*P* < 0.05), primarily reflecting the lower within-group dispersion of the SS samples. The bacterial phyla Proteobacteria, Firmicutes, Actinobacteria and Bacteroidota predominated in the microbiomes associated with spider mites and cultivation substrates, while distinct differences in microbial composition were detected between spider mites and between substrates under the two cultivation systems (Figure 1E). At the phylum level, Proteobacteria, Firmicutes, and Bacteroidota were the predominant contributors to microbiome divergence in spider mites between the two cultivation systems (Supplementary Figure 1C in Supplementary Information), whereas Proteobacteria, Actinobacteria and Cyanobacteria accounted for the primary differences in substrate-associated microbial communities (Supplementary Figure 1D in Supplementary Information). Furthermore, genus-level analyses identified specific bacteria that were differentially abundant between the two cultivation systems in both the spider mites (Figure 1F) and the growth substrates (Figure 1G).

### 3.2 Distinct spider mite microbiomes are associated with different cultivation systems

**Figure 2:**
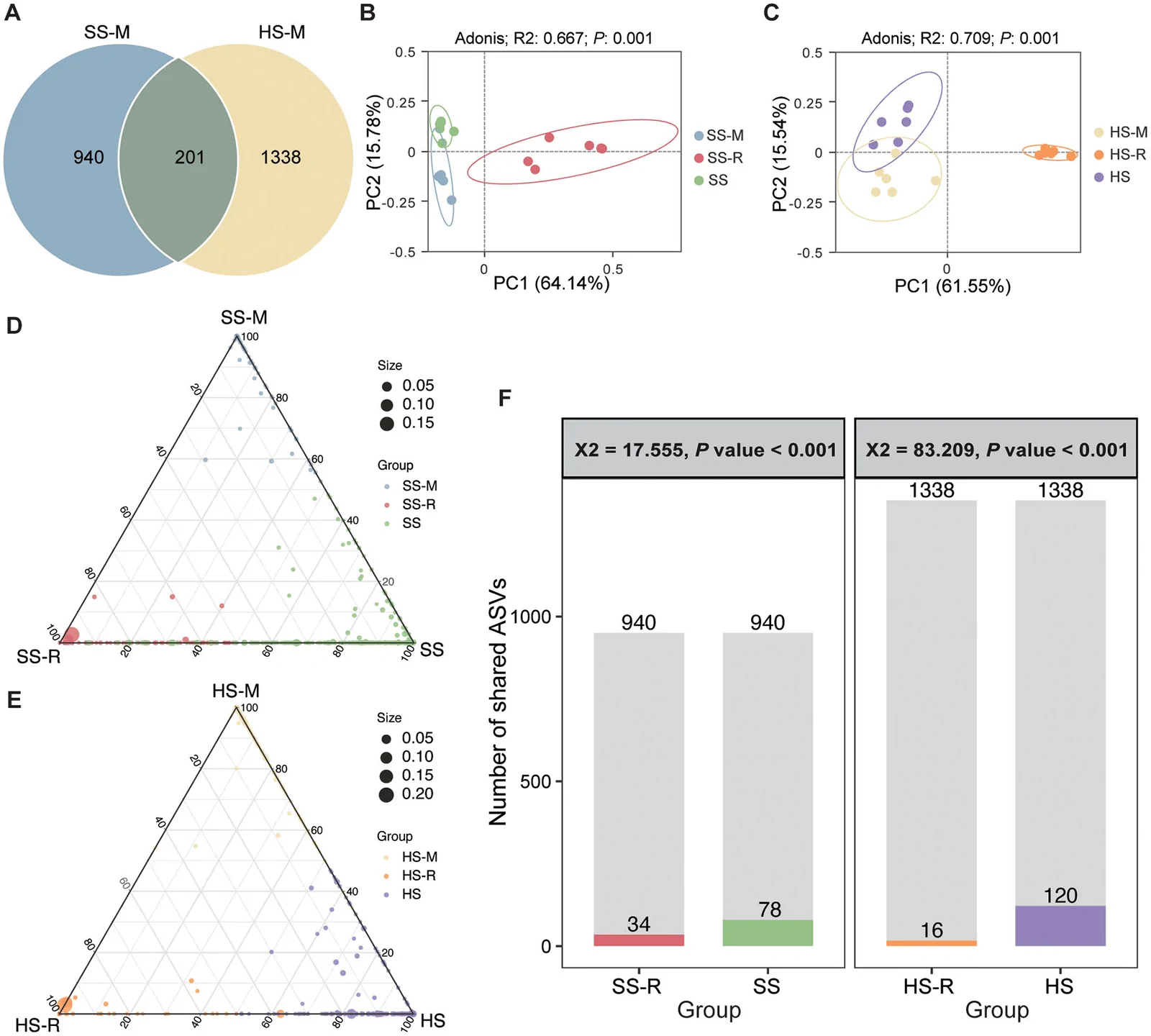
Comparative analysis of bacterial communities associated with spider mites *T. pueraricola*, plant roots and cultivation substrates under distinct conditions. **(A)** Venn diagram of shared and unique ASVs between spider mites reared on common bean plants under soil and hydroponic system. (**B**, **C)** PCoA analyses based on weighted UniFrac dissimilarities illustrating differences in microbial community among spider mites, roots, and cultivation substrates in soil system (**B**) and hydroponic system **(C)**. R^2^ and *P* values are based on an Adonis test. (**D, E)** Ternary plots of bacterial ASVs shared among spider mites, roots, and cultivation substrates in soil system (**D**) and hydroponic system (**E**). Each symbol represents a single ASV with symbol size corresponding to its relative abundance, and symbol position indicating the relative contribution to the total relative abundance. **(F)** Bar plots showing ASVs overlaps between spider mites and plant roots or cultivation substrates. In soil system, 34 and 78 ASVs out of the 940 mite-specific ASVs were detected in plant roots (SS-R) and soil substrates (SS), respectively; 16 and 120 out of the 1,338 mite-specific ASVs in the hydroponic system were shared with plant roots (HS-R) and hydroponic substrates (HS), respectively. Proportional differences within group was assessed using Pearson's chi-squared test, and a two-tailed *P* < 0.05 was considered statistically significant. SS-R, plant root in soil system; HS-R, plant root in hydroponics system.

To determine the extent of microbial divergence, we first compared bacterial ASVs in spider mites across the two systems. A mere 201 bacterial ASVs were shared between both groups, with the majority being unique to respective system (Figure 2A). To further examine potential associations underlying these microbial differences, we compared the bacterial community compositions of spider mites, plant roots and their respective substrates. Mite-associated microbial communities clustered more closely with their respective cultivation substrates than with the plant roots, as demonstrated by principal coordinate analysis (PCoA) (Figures 2B, C). Moreover, a subset of mite-specific ASVs overlapped with those in the corresponding cultivation substrates (Figures 2D, E). For example, we found that soil-reared spider mites and their corresponding substrate shared several nitrogen-cycling bacteria, including *Candidatus Nitrocosmicus, Mesorhizobium* and *Nitrospira*, and *Nitrospira* was detected in both mites and the substrate within the hydroponic system (Supplementary Table 1 in Supplementary Information). Notably, the extent of this overlap was significantly greater than that observed between spider mites and roots in soil system (Figure 2F, Supplementary Figure 2A in Supplementary Information) and hydroponic system (Figure 2F, Supplementary Figure 2B in Supplementary Information). These results suggest that spider mite microbiomes exhibit closer compositional associations with cultivation substrates than with root-associated microbial communities. Furthermore, shared bacteria between spider mites and soil substrate were dominated by Firmicutes, Actinobacteriota and Proteobacteria (Supplementary Figure 2C in Supplementary Information), while those shared between spider mites and hydroponic substrate were enriched in Firmicutes and Bacteroidota at the phylum level (Suppelemtary Figure 2D and Supplementary Table S2 in Supplementary Information).

### 3.3 Cultivation systems correlate with distinct metabolic profiles in plant leaves

**Figure 3:**
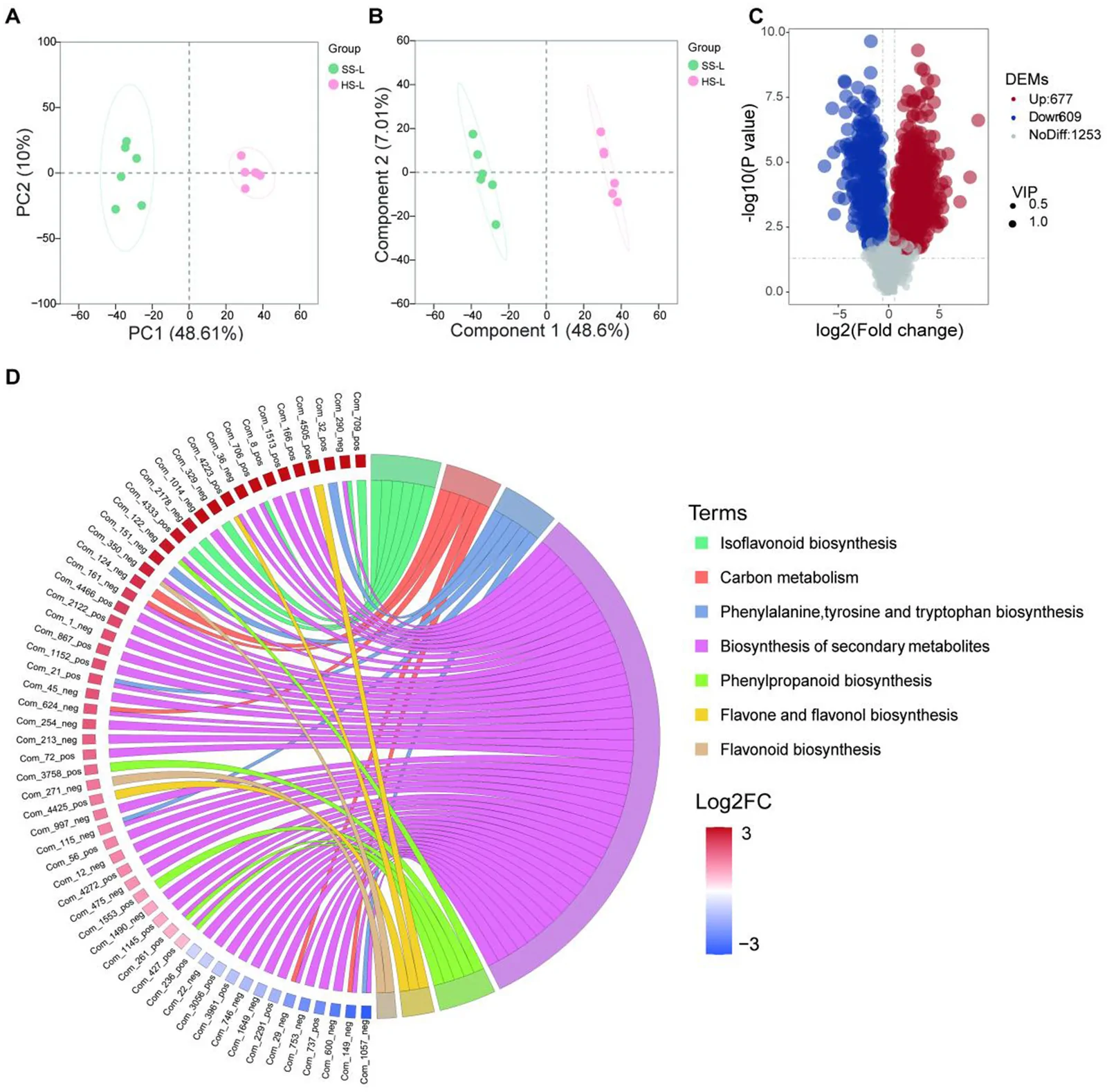
Metabolomic analyses of common bean leaves grown in different cultivation systems. **(A**, **B)** Principal component analysis (PCA, **A**) and partial least squares discriminant analysis (PLS-DA), **(B)** were performed on log^2^-transformed data with standard scaling (unit variance autoscaling) preprocessing, showing distinct metabolic profiles of leaves under soil and hydroponic systems. **(C)** The volcano map of differential expressed metabolites (DEMs) in common bean leaves from different cultivation systems. **(D)** Chord diagram visualizing the enrichment of DEMs in key KEGG metabolic pathways. The ribbons connect specific metabolites ID to their corresponding biological terms, with the color scale indicating the log2FC. SS-L, plant leaf in soil system; HS-L, plant leaf in hydroponics system.

Leaf metabolites of the common bean under different cultivation systems were profiled using liquid chromatography-mass spectrometry (LC-MS), yielding a total of 2,539 detected metabolic features, mainly including lipids and lipid-like molecules (31.7%), organic acids and derivatives (18.5%), phenylpropanoids and polyketides (13.1%), organoheterocyclic compounds (12.5%), organic oxygen compounds (10.6%), benzenoids (7.8%), nucleosides, nucleotides and analogs (1.6%), organic nitrogen compounds and alkaloids and derivatives (1.3%) (Supplementary Figure 3A in Supplementary Information). Multivariate statistical analyses, including PCA revealed a different metabolic profiles, with samples clustering distinctly according to their cultivation substrate (Figure 3A). The PLS-DA model showed clear discrimination between the two cultivation systems (Figure 3B). Seven-fold cross-validation indicated excellent model fit and predictive performance, permutation testing (200 permutations) also showed that the model was not overfitted and that the observed group separation was statistically reliable (Supplementary Figure 4 in Supplementary Information). Following Benjamini–Hochberg correction for multiple testing, metabolites with VIP > 1, FDR-adjusted *P* < 0.05, and FC ≥ 1.5 or FC ≤ 0.7 were considered differentially expressed. A total of 1,286 differential metabolites were identified (Supplemenatary Table 3 in Supplementary Information), with 677 upregulated and 609 downregulated in hydroponically grown leaves relative to soil-grown leaves (Figure 3C). These differential metabolites were assigned to functional modules including metabolism, genetic information processing, and environmental information processing through KEGG pathway annotation (Supplementary Figure 3B in Supplementary Information). Notably, a subset of differential metabolites were significantly enriched in the pathways of isoflavonoid biosynthesis and carbon metabolism (Figure 3D, Supplementary Table 4 in Supplementary Information). In addition, other differentially expressed metabolites mapped onto a series of interconnected metabolic pathways, such as phenylalanine, tyrosine and tryptophan biosynthesis, phenylpropanoid biosynthesis, flavone and flavonol biosynthesis, and flavonoid biosynthesis (Figure 3D, Supplemenatry Table 4 in Supplementary Information). To better understand the nutritional status of the host plants, we further analyzed the primary metabolites in the leaf metabolome. KEGG pathway enrichment analysis revealed that nitrogen metabolism (map00910) was significantly altered between the two cultivation modes. Moreover, key amino acids, including L-Asparagine and L-Glutamine, exhibited significantly higher accumulation in hydroponically grown leaves than in soil-grown leaves. In contrast, citric acid, a key organic acid involved in the tricarboxylic acid (TCA) cycle, was significantly down regulated in hydroponic leaves. These results indicate that hydroponically grown plants possess a primary nutritional profile, characterized by active nitrogen assimilation and amino acid accumulation, and cultivation substrates trigger metabolic reprogramming in common bean leaves, potentially mediating the growth-defense trade-off via the modulation of carbon metabolism toward defensive secondary metabolites, which subsequently influences herbivore performance.

### 3.4 Cultivation substrate drives multi-scale associations among substrate microbiome and leaf metabolome and mite microbiome

**Figure 4:**
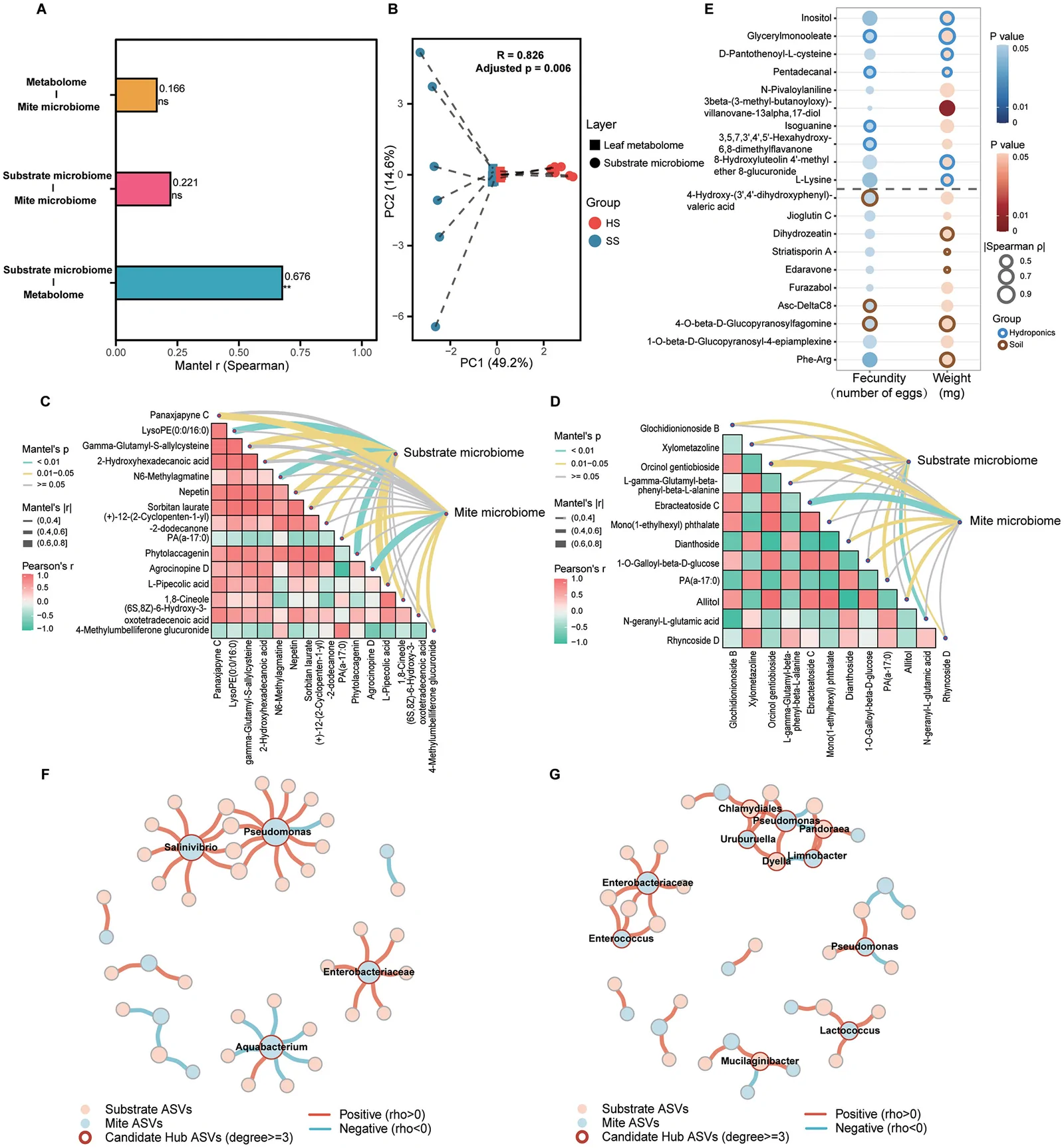
Cross-omics associations across cultivation systems. (**A)** Mantel correlation among among substrate microbiome, leaf metabolome and insect microbiome. Asterisks indicate significance levels (**P < 0.01; ns, not significant). (**B)** Procrustes superimposition of substrate microbiome (circles) and leaf metabolome (squares) in soil (blue) and hydroponic (red) systems. Dashed lines connect paired samples. Mantel test analysis reflecting the correlations among plant metabolites, substrate microbiomes and spider mite microbiomes in soil **(C)** and hydroponic **(D)** cultivation systems. The color gradient in the heatmap represents Pearson's correlation between metabolites, line thickness indicates the absolute value of Mantel's r and line color indicates Mantel's *P* value. **(E)** Bubble plot showing the Spearman rank correlations calculated within each cultivation system between core metabolites and fecundity (left column) or body weight (right column). Metabolites are ordered by enrichment direction, with hydroponic-enriched metabolites above the dashed line and soil-enriched metabolites below. Bubble size represents the absolute value of Spearman's rank correlation coefficient. Bubble fill color represents *P* values (Fecundity: blue gradient; Weight: red gradient). Bubble outline color indicates the cultivation system from which the displayed the stronger correlation originated (brown indicates soil, blue indicates hydroponic).Association network linking substrate microbiome and insect microbiome in soil **(F)** and hydroponic **(G)** cultivation systems. Each network showing the top 40 candidate paired ASVs associations ranked by Pearson correlation strength. Pink nodes represent substrate ASVs and blue nodes represent mite ASVs. Red and blue edges indicate positive and negative correlations, respectively. Node size is proportional to the square root of node degree. ASVs with degree ≥ 3 are labeled in bold with dark red borders as high-degree candidate nodes.

A significant distance-based correlation between the substrate microbiome and the leaf metabolome was detected by Simple Mantel tests (*r* = 0.676, adjusted *P* = 0.003; Figure 4A), whereas correlations involving the mite microbiome were weak and non-significant (all adjusted *P* > 0.05; Figure 4A). This pattern was corroborated by Procrustes analysis, which showed high concordance between the substrate microbiome and leaf metabolome ordinations (Procrustes r = 0.826, adjusted *P* = 0.006; Figure 4B). However, partial Mantel tests controlling for cultivation group reduced all three pairwise correlations to non-significance (Supplementary Table 5 in Supplementary Information), indicating that the observed concordance between the substrate microbiome and leaf metabolome is a consequence of cross-layer responding in parallel to the cultivation substrate. Random Forest regression further identified several important metabolites that showed with significant Mantel correlations (*P* < 0.05) with substrate or mite microbiome in both systems, such as 1,8-Cineole, l-pipecolic acid, agrocinopine D, lysoPE(0:0/16:0), nepetin, phytolaccagenin, 1-O-Galloyl-beta-D-glucose, ebracteatoside C (Figures 4C, D, Supplemenatry Table 6 in Supplementary Information). We also identified 20 core metabolites that were both highly discriminating between soil and hydroponic systems, including 10 hydroponic-enriched and 10 soil-enriched metabolites (Figure 4E). Spearman correlations between each core metabolite and fitness traits independently within each cultivation system showed that three metabolites with *P* < 0.05 and |ρ| ≥ 0.6 for fecundity (Inositol, ρ = 0.83; L-Lysine, ρ = −0.83; Phe-Arg, ρ = −0.84) and one metabolite for body weight (3beta-(3-methyl-butanoyloxy)-villanovane-13alpha,17-diol, ρ = 0.94) (Supplemenatry Table 7 in Supplementary Information). Cross-domain substrate-mite ASV networks showed that the soil network was more densely connected, featuring prominent candidated nodes including *Pseudomonas, Salinivibrio*, and Enterobacteriaceae (family level), which formed extensive positive-correlation subnetworks (Figure 4F, Supplemenatry Table 8 in Supplementary Information). By contrast, the hydroponic network showed isolated components (Figure 4G). Candidated nodes in the mite ASVs mainly included Enterobacteriaceae (family level), *Enterococcus, Pseudomonas, Lactococcus, Uroburuella*, while substrate-associated hubs comprised *Pandoraea, Mucilaginibacter*, Chlamydiales (order level) and *Dyella* (Figure 4G, Supplemenatry Table 8 in Supplementary Information). Although the taxonomic identities of hub nodes differ substantially between two systems, consistent with cultivation-driven community turnover.

## 4 Discussion

### 4.1 Cultivation substrates may represent important environmental sources of herbivore-associated microbiota

Cultivation substrates serve as a primary environmental reservoir that directly links to aboveground herbivore microbiome. The bacterial community of spider mites demonstrated high responsiveness to changes in the host plant growth substrate, with significantly more bacterial ASVs shared with the substrate than with the plant roots (Figure 2). Although *T. pueraricola* is a foliar-feeding herbivore, increasing evidence underscores that the gut microbiomes of aboveground insects are not maintained solely through dietary intake, but can be acquired directly from the surrounding belowground or soil environment (Kikuchi et al., 2007; Gomes et al., 2020; Shan et al., 2024). This ecological pattern is highly consistent with previous observations by Hannula et al. (2019), who demonstrated that the microbiomes of foliar-feeding caterpillars on intact plants more closely resembled soil microbiomes than leaf microbiomes. Such parallels strongly support the notion that belowground microbial reservoirs play an important role in shaping the microbiomes of aboveground foliar-feeding herbivores (Hooper and Gordon, 2001; Frago et al., 2012; Etalo et al., 2018; Pineda et al., 2013). Since elucidating the tripartite interactions among the substrate, phyllosphere and herbivore microbiomes is critical for fully understanding microbial assembly mechanisms, the present study did not analyse of leaf-associated microbial communities. Therefore, integrating phyllosphere microbiome profiling into future studies will be necessary to clarify the potential contributions of leaf-associated microbes to spider mite microbiome assembly. Furthermore, the comparison between soil and hydroponic systems inevitably involves multiple environmental differences, including nutrient availability, water conditions, physical properties and microbial inputs, as these factors may collectively modulate the multi-trophic effects from belowground environments to aboveground herbivore fitness. While our study highlights a multi-trophic association, the individual contributions of microbial and abiotic factors require further investigation, such as through sterile-substrate experiments or microbial transplantations. Future studies using controlled manipulations of individual environmental factors will be necessary to disentangle their relative contributions to plant metabolism, microbial assembly and herbivore fitness.

### 4.2 Substrate-driven metabolic reprogramming in leaves alters spider mite fitness

Hydroponic cultivation reprogrammed leaf metabolism by enhancing defense-related secondary metabolism while maintaining favorable nutritional status, which may explain the contrasting effects on spider mite fitness. Aromatic amino acids are central precursors for defensive compounds, including phenylpropanoids and flavonoids (Vogt, 2010; Tohge et al., 2013; Maeda, 2019), and play key roles in plant defense against herbivores (Moormann et al., 2022; Liu L. et al., 2021; Li et al., 2025). In our study, hydroponically grown common bean leaves showed coordinated increases in shikimate pathway intermediates, including quinic acid, shikimic acid, indole, and 3-hydroxybenzoic acid, together with numerous downstream metabolites involved in phenylpropanoid, flavonoid, isoflavonoid, flavone and flavonol biosynthesis (Figure 3D, Supplemenatry Table 4 in Supplementary Information). Many of these metabolites are well known to reduce herbivore feeding, growth and survival (Zhang X. et al., 2022; Piubelli et al., 2003; Diaz Napal and Palacios, 2015; Punia et al., 2023; Wang et al., 2006), suggesting that hydroponic cultivation enhanced the chemical defenses of common bean leaves. At the same time, changes in primary metabolism were consistent with increased investment in defense. Increased levels of D-gluconate, glycerate and butanoyl-CoA, together with reduced citrate and malate, suggest that carbon flux was redirected from the tricarboxylic acid (TCA) cycle toward the shikimate pathway to support secondary metabolite biosynthesis. Metabolomic evidence also indicates that hydroponic leaves were unlikely to be nitrogen deficient, as the nitrogen metabolism pathway was enriched and both L-asparagine and L-glutamine accumulated. This nutritional profile is consistent with the lower abundance of nitrogen-fixing bacteria in mites feeding on hydroponic leaves, suggesting that nitrogen-rich host tissues may reduce the dependence of mites on symbiotic nitrogen fixation. A recent study showed that low nitrogen restricts brown planthopper performance by simultaneously reducing amino acid availability and increasing defensive flavonoids (Chen et al., 2026). Therefore, our results suggest that the higher amino acid availability in hydroponic leaves may support increased spider mite fecundity, whereas enhanced chemical defenses may impose physiological constraints that restrict body weight. Future studies should directly quantify total nitrogen and free amino acids to validate the nutritional status inferred from the metabolomic data. Moreover, the associations among metabolites, microbial communities and spider mite fitness observed in this study provide a foundation for understanding their tripartite interactions. Next experimental work, involving manipulation of candidate metabolites, is essential to validate their functional roles in modulating spider mite performance.

### 4.3 Cultivation system shapes nitrogen-cycling bacteria and plant metabolism linked to spider mite fitness

Symbiotic bacteria play a key role in the ecological adaptation of herbivores (Douglas, 2015; Zhang Y. et al., 2022; Jang et al., 2024; Cheng et al., 2017). Nitrogen-fixing bacteria have been reported as symbionts across various insect orders, including Coleoptera, Diptera and Hemiptera, where taxa such as the Rhizobiales facilitate nitrogen assimilation in insects (Bar-Shmuel et al., 2020). These bacteria are involved in different processes of the nitrogen cycle. *Candidatus Nitrocosmicus* catalyzes ammonia oxidation, *Nitrospira* is responsible for nitrite oxidation (Liu W. et al., 2021; Zhang et al., 2023; Zhao et al., 2025), and *Mesorhizobium*, a key nitrogen-fixing bacterium in the Rhizobiales, provides nitrogen to plants through nitrogen fixation (Laranjo et al., 2014; Kenasa et al., 2018). In the soil cultivation system, both spider mites and their corresponding substrates harbored nitrogen-cycling bacteria, including *Candidatus Nitrocosmicus, Mesorhizobium* and *Nitrospira*, whereas only *Nitrospira* was detected in spider mites from the hydroponic system (Supplemenatry Table 1 in Supplementary Information). This pattern suggests that the hydroponic system was associated with a simplified nitrogen-cycling microbial network. Because nitrogen availability influences both primary metabolism and the biosynthesis of defensive compounds (Chen et al., 2023), differences in nitrogen-cycling microorganisms may affect nutrient availability in the cultivation system and thereby influence leaf nutritional quality. Combined with the enhanced defense-related metabolism observed in hydroponically grown leaves, these changes may explain the contrasting fitness responses of spider mites. Although spider mite growth was suppressed under the hydroponic system, fecundity increased significantly, the contrasting changes in body mass and fecundity likely represent independent phenotypic responses of *T. pueraricola* to nutrient limitation and strengthened plant chemical defenses. Such adaptive strategies are commonly observed in arthropods, in which limited resources are preferentially allocated to reproduction to maintain population persistence under stressful or resource-constrained conditions (Awmack and Leather, 2002; Boggs, 2009; Zera and Harshman, 2001; Stadler, 1995). Future studies should further investigate plant nitrogen content and examine nitrogen metabolism in spider mites to clarify the contribution of nitrogen availability to these responses. Collectively, these findings suggest that cultivation system-driven changes in nitrogen-cycling microbial communities and plant metabolism are associated with variation in spider mite nutritional adaptation and fitness responses. Although these analyses revealed potential links among microbiota, plant metabolism, and spider mite performance, functional validation of key microbial ta xaremains necessary. Future studies integrating larger-scale sampling and targeted microorganisms will help clarify the mechanisms underlying these interactions.

## 5 Conclusion

Our study demonstrates that cultivation substrate concomitantly shapes the leaf metabolome, substrate microbiome, and spider mite microbiome, with all three omics layers diverging between soil and hydroponic systems. Distance-based association analyses showed substrate-associated co-divergence, and substrate-mediated metabolic shifts further influence herbivore performance. These results provide theoretical insights for optimizing agricultural ecosystems and advancing sustainable pest control strategies. Future investigations with larger sample sizes and functional validation should focus on the mechanistic basis of substrate-metabolite-mite interactions, particularly the roles of nitrogen-cycling bacteria and defense-associated metabolites in shaping herbivore microbiome and fitness.

## Data Availability

The original contributions presented in the study are publicly available. This data can be found here: NCBI (https://www.ncbi.nlm.nih.gov/), BioProject accession: PRJNA1438036. SRA accession numbers: SRR37652997-SRR37653032.
