# Supplementary material for: Cultivation substrates alter plant metabolic profiles with concomitant impacts on herbivore performance
Source: Front Microbiol. 2026 Sep 1;17:1893579. doi: 10.3389/fmicb.2026.1893579 (PMC13574806; doi:10.3389/fmicb.2026.1893579)
Supplement: Supplementary file 1 [file Table_1.docx]

**Supplemental table 1**. Participant demographics, ophthalmic baseline characteristics, and surgical outcomes.

| No.Patient | Sex | Age, years | Eye |  | BCVA, LogMAR (Snellen) | |  | AL,mm | | |  | OCT findings | | | | | | |
| --- | --- | --- | --- | --- | --- | --- | --- | --- | --- | --- | --- | --- | --- | --- | --- | --- | --- | --- |
|  |  |  |  |  |  |  |  |  |  |  |  |  |  |  |  |  |  |  |
|  |  |  |  |  | Pre-op | Last follow up |  | Pre-op | Post-op 1week | Last follow up |  | Pre-op | |  | Post-op, last follow up | | | |
| Pre-op | | | |  |  |  |  |  |  |  |  | MRS involving the fovea | MRS involved meridians |  | MRS involving the fovea | MRS involved meridians | MRS resolution | Follow up, mos |
| P1 | M | 20 | OS |  | 0.70 (20/100) | 0.30 (20/40) |  | 31.64 | 29.39 | 30.68 |  | Yes | 12/12 |  | No | 0 | Complete | 54 |
| P2 | M | 30 | OD |  | 3.00 (HM/BE) | 0.70 (20/100) |  | 32.41 | 28.71 | 30.12 |  | Yes | 12/12 |  | No | 0 | Complete | 80 |
| P3 | M | 25 | OD |  | 0.00 (20/20) | 0.00 (20/20) |  | 27.89 | 27.15 | 27.86 |  | Yes | 12/12 |  | No | 0 | Complete | 68 |
|  |  |  | OS |  | 0.00 (20/20) | 0.00 (20/20) |  | 27.60 | 26.81 | 27.57 |  | Yes | 11/12 |  | No | 6/12 | Partial | 68 |
| P4 | F | 22 | OS |  | 0.10 (20/25) | -0.08 (20/16) |  | 24.81 | 23.79 | 24.33 |  | Yes | 12/12 |  | No | 0 | Complete | 29 |
| P5 | F | 25 | OS |  | 0.82 (20/125) | 0.82 (20/125) |  | 31.03 | 30.74 | 31.75 |  | Yes | 12/12 |  | No | 6/12 | Partial | 102 |
| P6 | F | 28 | OD |  | -0.08 (20/16) | 0.00 (20/20) |  | 27.63 | 27.40 | 27.77 |  | No | 4/12 |  | No | 0 | Complete | 110 |
| P7 | F | 23 | OD |  | 0.22 (20/32) | 0.22 (20/32) |  | 31.61 | 29.55 | 31.50 |  | No | 10/12 |  | No | 0 | Complete | 60 |
| P8 | F | 26 | OD |  | 0.10 (20/25) | 0.00 (20/20) |  | 29.76 | 29.44 | 30.63 |  | No | NA |  | No | 0 | Complete | 108 |
|  |  |  | OS |  | 0.10 (20/25) | 0.00 (20/20) |  | 28.90 | 28.25 | 30.15 |  | Yes | NA |  | No | 0 | Complete | 108 |
| P9 | F | 24 | OD |  | 0.22 (20/32) | 0.10 (20/25) |  | 31.84 | 31.64 | 32.45 |  | No | 3/12 |  | No | 0 | Complete | 84 |
| P10 | M | 21 | OD |  | 0.82 (20/125) | 0.82 (20/125) |  | 31.10 | 29.42 | 31.78 |  | Yes | 12/12 |  | No | 7/12 | Partial | 86 |
| P11 | F | 23 | OS |  | 0.30 (20/40) | 0.10 (20/25) |  | 28.60 | 26.82 | 27.6 |  | No | 6/12 |  | No | 4/12 | Partial | 45 |
| P12 | M | 22 | OS |  | 0.00 (20/20) | 0.00 (20/20) |  | 25.79 | 23.68 | 24.98 |  | Yes | 12/12 |  | No | 0 | Complete | 96 |
| P13 | M | 23 | OD |  | 0.22 (20/32) | 0.00 (20/20) |  | 29.47 | 28.56 | 29.64 |  | No | 9/12 |  | No | 0 | Complete | 75 |
| P14 | M | 24 | OD |  | 0.22 (20/32) | 0.22 (20/32) |  | 30.38 | 28.08 | 29.87 |  | No | 6/12 |  | No | 2/12 | Partial | 66 |
| P15 | M | 18 | OD |  | 0.10 (20/25) | 0.00 (20/20) |  | 26.80 | 25.80 | 27.13 |  | Yes | 12/12 |  | No | 6/12 | Partial | 90 |
|  |  |  | OS |  | 0.00 (20/20) | 0.00 (20/20) |  | 26.92 | 25.64 | 26.95 |  | Yes | 12/12 |  | No | 3/12 | Partial | 79 |
| P16 | F | 26 | OD |  | 0.00 (20/20) | 0.00 (20/20) |  | 29.43 | 28.07 | 28.51 |  | No | 13/18 |  | No | 7/18 | Partial | 60 |
| P17 | M | 19 | OD |  | 0.22 (20/32) | 0.10 (20/25) |  | 31.33 | 30.84 | NA |  | No | 12/18 |  | No | 3/18 | Partial | 28 |
| P18 | F | 14 | OS |  | 0.10 (20/25) | 0.10 (20/25) |  | 30.54 | 28.69 | NA |  | No | 10/18 |  | No | 5/18 | Partial | 49 |
| P19 | F | 22 | OD |  | 0.00 (20/20) | 0.00 (20/20) |  | 28.16 | 26.28 | 27.86 |  | Yes | 12/12 |  | No | 5/18 | Partial | 31 |
|  |  |  | OS |  | 0.00 (20/20) | 0.00 (20/20) |  | 27.78 | 25.38 | 26.67 |  | Yes | 12/12 |  | No | 0 | Complete | 31 |
| P20 | M | 18 | OD |  | 0.10 (20/25) | 0.30 (20/40) |  | 33.67 | 32.28 | 34.71 |  | No | 5/18 |  | No | 16/18 | No | 35 |
|  |  |  | OS |  | 0.10 (20/25) | 0.30 (20/40) |  | 33.97 | 31.01 | 33.46 |  | No | 17/18 |  | No | NA | NA | 35 |
| P21 | M | 14 | OD |  | 0.10 (20/25) | 0.10 (20/25) |  | 29.31 | 28.28 | 29.62 |  | No | 11/18 |  | No | 6/18 | Partial | 79 |
|  |  |  | OS |  | 0.10 (20/25) | 0.10 (20/25) |  | 30.18 | 29.00 | 30.25 |  | No | 13/18 |  | No | 13/18 | No | 79 |

AL, axial length; BCVA, best-corrected visual acuity; LogMAR, logarithm of the minimum angle of resolution; MRS, macular retinoschisis; NA, not available; OCT, optical coherence tomography; OD, oculus dexter; OS, oculus sinister. Highlighted in red were those with 5.00 ± 1.25 years of postoperative follow-up.

*In patient P21, the right eye had 5/18 MRS involved meridians and the left eye had 6/18 at the 5-year follow-up. By 79 months postoperatively, the extent of schisis had increased in both eyes (see above).
