# Supplementary material for: Cultivation substrates alter plant metabolic profiles with concomitant impacts on herbivore performance
Source: Front Microbiol. 2026 Sep 1;17:1893579. doi: 10.3389/fmicb.2026.1893579 (PMC13574806; doi:10.3389/fmicb.2026.1893579)
Supplement: Supplementary file 2 [file Table_2.docx]

Supplementary Material

# Supplementary Figures and Tables

## Supplementary Figures

**Supplementary Figure 1.** **Comparative analysis of bacterial diversity in the substrate and spider mite microbiomes under different cultivation systems. A** Box plots showing Chao1 diversity of bacterial communities in spider mites across different systems. **B** Box plots showing Chao1 diversity of different cultivation substrates. Statistical significance determined by the Wilcoxon rank-sum test, “ns” indicates not significant. **C, D** SIMPER analyses based on Bray-Curtis dissimilarity identifying the top 10 phylum-level bacteria contributing to the community dissimilarity of spider mites (**C**) or cultivation substrates (**D**) under different systems. Bubble size represents the relative abundance of the species, the contribution rate of each species to the dissimilarity between the two groups is indicated by the corresponding values.

**Supplementary Figure** **2. ASVs comparison and bacterial phylum composition among spider mite, plant root and cultivation substrate across soil and hydroponic systems. A, B** Venn diagrams showing the shared or unique ASVs among mite-specific microbial communities, plant roots and cultivation substrates under soil system (**A**) and hydroponic system (**B**). **C, D** Pie charts depicting the relative abundance and diversity of bacterial phyla for ASVs shared between mite-specific communities and their respective substrates under soil system (**C**) and hydroponic system (**D**). Numbers in parentheses represent the percentage of each phylum. SS-M-uni, 940 ASVs only exists in spider mite under soil system, HS-M-uni, 1338 ASVs only exists in spider mite under hydroponic system.

**Supplementary Figure 3. Classification and functional annotation of differentially expressed metabolites (DEMs) in spider mites under the two cultivation systems.** **A** Pie chart showing the proportion of different categories in total differential metabolites. **B** KEGG pathway classification of the DEMs. The bar plot displays the enrichment of metabolites across three primary functional categories: metabolism (orange), genetic information processing (blue), and environmental information processing (red). Numbers and percentages adjacent to the bars indicate the count and proportion of metabolites assigned to each sub-pathway.

**Supplementary Figure 4. Partial least squares discrimination analysis. A** PLS-DA permutation test (200 permutations). R² and Q² regression lines versus correlation between permuted and original Y; Q² intercept < 0 confirms no overfitting. **B** PLS-DA score plot. Samples projected onto the first two components with 95% confidence ellipses; R²Y and Q²Y values indicate model fit and predictive ability, respectively.

# Supplementary Tables

**Supplementary Table 1.** Numbers of three nitrogen-fixing bacteria in substrates, plant roots, and spider mites under different cultivation systems.

**Supplementary Table 2.** List of bacterial phyla for ASVs shared between mite-specific communities and their respective substrates.

**Supplementary Table 3.** List of 1286 differential expressed metabolites (DEMs) in common bean leaves from different cultivation systems.

**Supplementary Table 4.** The enrichment of DEMs in key KEGG metabolic pathways.

**Supplementary Table 5.** Data summary of Mantel and partial Mantel test.

**Supplementary Table 6.** Important metabolisms assocoated with substrate and mite microbiomes in soil and hydroponic cultivation systems.

**Supplementary Table 7.** Complete raw and FDR-adjusted statistics for testing correlations between metabolites and mite fitness in two systems.

**Supplementary Table 8.** Statistics of association network linking substrate-mite ASVs in soil and hydroponic cultivation systems.
